# Taxonomy for citizen actions on public health and climate change: a proposal

**DOI:** 10.11606/s1518-8787.2021055003823

**Published:** 2021-12-16

**Authors:** Lidice Álvarez-Miño, Robinson Taboada Montoya

**Affiliations:** I Universidad del Magdalena Health Sciences Faculty Santa Marta Magdalena Colombia Universidad del Magdalena. Health Sciences Faculty. Santa Marta, Magdalena, Colombia

**Keywords:** Classification, Public Health, Climate Change, Citizen Science

## Abstract

Facing complex issues such as climate change and its effects on public health require the participation of various actors. The research tool citizen science is one way for people to get involved. Through it, citizens collaborate with scientists to find solutions to problems in their territories. From a participatory work with citizens, we designed a taxonomy proposal, which can facilitate citizen and community action in suggesting research ideas. We expect stakeholders to use it to systematically classify and code initial questions and answers on public health and climate change issues. The development of this taxonomy integrates the global agenda of Sustainable Development Goals (SDG) in such a way that citizens not only help their communities but also, the direct fulfillment of SDGs such as Climate Action (SDG 13), indirectly impacting other SDGs — given their interdependent nature (SDG 3, SDG 5, SDG 6, SDG 7, SDG 11, SDG 12). The systematic classification and coding of citizens’ contributions worldwide will contribute to the large-scale organized collection of information to be analyzed in proposing better responses to reduce the impacts of climate change on health.

## INTRODUCTION

Climate change is a complex terrestrial phenomenon of periodic alterations of local and regional weather patterns. However, the current climate change is the result of the excessive concentration of greenhouse gases (GHG), mainly CO_2_, emitted by the production and consumption activities of our human species^1^. It impacts all aspects of humanity in some way. Thus, millions face serious health and well-being issues, especially vulnerable groups such as children^2^, the elderly^3^, people with non-communicable diseases, inhabitants of coastal cities, and those in poverty^4^.

Increasingly more extreme and frequent meteorological events affect public health directly^5^. Evidence shows mortality rises during the hottest days of the year^6^, especially among those with non-communicable diseases such as diabetes mellitus^7^. If we fail to adapt, we expect a significant rise in mortality due to heatwaves in tropical and sub-tropical countries by 2080^8^. The impact of floods on health depends on geography, demographics and environmental urbanization^9^. However, in the short term, disaster victims suffer a rise of 50% in the mortality rate for up to a year after the event, and endure great psychological distress and outbreaks of infectious diseases due to poor sanitary conditions^10^.

On the other hand, natural systems mediate how climate change affects health, such as water-borne diseases and vectors. Variations on rain and drought patterns change the concentration of microorganisms in the available bodies of water, altering diarrhea frequency^11^, especially in children under the age of five^12^. Changes in meteorological variables such as rain and humidity, and higher temperatures alter vector dynamics, exposing people to diseases transmitted via vectors and zoonoses whose presence and proliferation was impossible^13^. Another such effect includes air quality, especially due to chronic exposition to ozone (O_3_) in urban areas following the higher global temperature^17^.

Humans institutions and systems also impact people’s health. We highlight syndemics, such as the global epidemic of obesity, malnutrition and climate change. Business models, food systems, civil societies, national, and international governments have disturbed the health of millions of people and of the planet^18^.

Climate change is also emerging as a threat to people’s mental well-being. Increasingly intense and frequent natural disasters contribute directly to the reduced satisfaction in the lives of affected communities^19^, and exposure to them leads predominantly to post-traumatic stress disorder and depression. However, the depth of the impact depends on risk factors such as gender, socioeconomic status, the previous state of mental health, and community resilience, among others^20^.

Anthropogenic climate change indirectly affects mental and emotional health via subtle and progressive alterations of different meteorological phenomena. For example, Zerbini et al. identified a higher incidence of suicide by hanging during the hottest and brightest days in São Paulo, suggesting a correlation^21^. On the other hand, a study with the Inuit community of northern Canada highlights that emotions such as fear, sadness, anger, anxiety, as well as low self-esteem and a weakening of their culture are a consequence of changes in their land due to climate change^22^.

Thus, understanding how health relates to climate change is a highly complex issue challenging the proposal of solutions. These can be small and simple but can amount to substantial and satisfactory changes.

One framework to generate action proposals is the global agenda of the Sustainable Development Goals (SDG) which considers systemic and complex approaches to problems government agendas have failed to address via 17 objectives articulated among themselves. Therefore, SDG 17: Partnerships for the goals establishes that fulfilling the SDGs should first focus on people. This is why we must promote and seek cooperative citizenship to effectively manage any action against climate change effects (SDG 13: Take urgent action to combat climate change and its impacts)^23^. Progress should be made in having people and their communities as the main players in deciding what positively impacts their well-being and of their environment. Additionally, institutions, governments and academia should favor strategies and mechanisms that allow citizens to more actively submit, create and execute ideas and proposals for the adaptation to and mitigation of climate change while protecting the health of specific groups in particular continents.

Different approaches are gaining strength and promoting participation on different levels. Such is the case of citizen science. According to it, individuals learn to formulate research ideas that they execute along scientists or by themselves to solve issues within their communities^24^. However, shaping and organizing these ideas within a theoretical framework is complicated, especially if they relate to problems arising from the complex relation between health and climate change.

To facilitate the participation of more people in science and public decision-making, we propose a taxonomy for citizen action to classify and harmonize into categories and subcategories all ideas related to the protection of public health, and adapt to and mitigate anthropogenic climate change.

## Development of the Taxonomy Proposal

Protecting public health rests on two fundamental strategies: promotion and prevention. Health as a collective right means more than medical/sanitary care in a territory; it is the complex result of the interaction of multiple factors that create conditions for the good development of each individual^25^.

Like health, defining health promotion is very complicated. After more than 30 years, it is still far from the goal of “Health for all by the year 2000” of the Ottawa Charter of 1986. The document contains the most classic definition of health promotion: a strategy to provide people with the tools and means necessary to control their health. For them, the articulated work of multiple stakeholders is required to achieve minimum living conditions and the control over multiple factors impacting health and well-being (social determinants of health) of the majority of the population^26^. Health promotion considers three key pillars: governance as the synergic and multisectoral collaboration between governmental, non-governmental, and civil society entities^27^, which also includes individual and small-collective decisions to effectively implement public policies and/or good resource administration to execute ideas around an issue. Another category belongs to health settings and establishes the construction of an environment guaranteeing sustainable development and general well-being, starting with homes and families, expanding to streets, neighborhoods, townships, and developing in schools, businesses and cities. The last promotion category concerns health knowledge and education to motivate people to accept behavioral changes influencing their values, beliefs and customs to ensure and improve their well-being^28^. This subcategory includes actions organized in levels, in line with the subject receiving the information: 1) Information of and training for actions in the interest of life and health, 2) Transformation of practices via education, 3) Communal and participative investigation; these “levels” can overlap seamlessly allowing for a better communal appropriation of information and knowledge.

The second public health strategy is prevention. It seeks to have individuals take the necessary actions to avoid diseases by identifying and effectively intervening in the risk factors of pathological processes related to climate change or environmental disturbance^29^.

The categories and subcategories for public health are paired with response strategies for the current climate change: adaptation and mitigation. Adaptation includes the capacity of human and non-human systems to face the direct and indirect impacts of climate change. In this context, communities and their members must “adjust” to the changing environment^30^. The subcategories correspond to critical measures for global adaptation: protection of drinking water^31^, reduction of the impact of floods, drought management, and heatwave alerts^32^, among others. The second category references actions to diminish individual and communal greenhouse gas emissions, focusing on transforming habits, customs and norms to be more sustainable via the reduction of the carbon footprint from transportation and other daily activities (diet, consumption of goods and other services)^33,34^.

We highlight the classification proposal attempts to address the need for a simple organizing tool for the general public to participate in protecting and promoting health and the planet. It is an open and dynamic proposal in its beginnings and we hope it will be embraced, modified, reformed and adjusted to different needs.

We also hope that the tool promotes positive collective sentiments via cooperating actions towards a common objective: protecting human and planetary health^35^. At the same time, we want any stakeholder to understand that they can propose solutions to improve the current adverse global situation, contributing to the resilience of individuals and communities.

Considering the need to guide the general public towards concrete actions they can take from their territories, strengthening the exercise of citizens in response to climate change and public health, we codified the described categories and subcategories to begin defining actions. For this, we created a matrix in which categories of public health and climate change intersect each other.

The categories, as previously mentioned, correspond to strategies to promote health, prevent diseases, adapt to and mitigate climate change. However, categorization follows mainly the perspective of public health, since actions can promote the well-being of individuals, groups and the planet. The process followed was:

A double-entry table developed the coding system. Each cell with intersecting public health and climate change strategies has a five-character code allowing for a comprehensive classifying system. However, exclusivity is unintended, since classified objects can be in several categories at once. The first character of the code (first two capitalized letters) corresponds to health promotion [A] and disease prevention [B]; the second character references the categories within health promotion: governance, healthy environment, health communication [from 1 to 3], and prevention (primary disease prevention [1]); the third character is either adaptation or mitigation [number 1 or 2]; the fourth code character belongs to the subcategories within each public health category [from 1 to 4], and the final character corresponds to adaptation and mitigation subcategories [1 to 4] (Figure).

**Figure:**
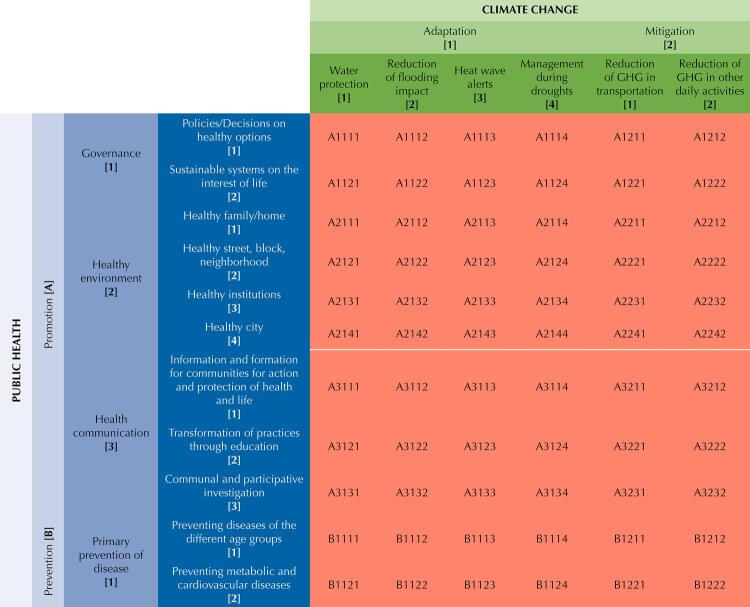
The codifying system for the proposal matrix.

GHG: greenhouse gases.

In an initial participatory exercise, we distributed the double-entry matrix to various people interested in the topic. They formulated research questions or proposed actions for the strategies, categories, and subcategories based on a contextualized problem. We placed the questions and the actions raised in the double-entry matrix eliminating the context or territory which participants analyzed. Thus, questions or actions are generalizable and adaptable, regardless of the specific community (neighborhoods, institutions, and cities).

Let us examine some of the code groups horizontally: The codes between A1111 - A1114 correspond to all possible research questions or actions framed within healthy policies for climate change adaptation strategies; questions on decision-making (at any level) intersecting water protection, the impact of floods, heatwave alerts, and drought management. The following two codes, A1211, and A1212 relate healthy policies to the reduction of greenhouse gases from transportation and daily life activities (mitigation).

For this first group of questions and actions, we show an example in Box 1. Box 2 has more questions and actions proposed by interested people, with their respective codes.

**Box 1:** Examples of some first questions and actions proposed to some stakeholders.

A. PROMOTION OF HEALTH AND CLIMATE CHANGE.	
A.1.1. Governance in health and adaptation to climate change.	
A.1.1.1.1. Policies/Decisions on health options – Water protection.	
Questions:	Actions:
*• How can we handle wastewater without risks?* *• How can we achieve permanent drinking water in the territory?*	*• Plans for oxidation ponds.* *• Subpoenas/fines for discarding tires in water sources.* *• Waste collection to raise awareness for the care of oceans and rivers.* *• Legislation and actions to protect moorlands, headwaters and water deposits via the collective protection of ecosystems.*
A.1.1.1.2. Policies/Decisions on health options – Reduction of the impact of floods.	
Questions:	Actions:
*• How can we create the infrastructure and education to eliminate mosquito breeding grounds in stagnant water during rainy seasons?* *• Which citizen actions would push decision-makers to reduce the probability of floods given the current climate variability?*	*• Sewers for draining wastewater.* *• Green urbanism.* *• Economic incentives for populations that reduce and safely handle waste in floodplains (for example, via taxes, or reducing utility fees).* *• Decent plans for population relocation.*
A.1.1.1.3. Policies/Decisions on health options – Heatwave alerts.	
Questions:	Actions
*• How can we design a health surveillance plan to face heatwaves?* *• Are heatwave early warning systems prioritized in policies to protect the health and life of the population?*	*• Planting trees that will increase the local topsoil.* *• Including construction standards with green roofs/walls in legislation.*
A.1.1.1.4. Policies/Decisions on health options – Drought management.	
Questions:	Actions:
*• What infrastructure and actions allow for a safe and efficient collection of rainwater in the territories?* *• Which local policies help save water?* *• Which productive processes can be modified to reduce water use?*	*• Planning the distribution of drinking water during crises.*
**A.1.2. Governance in health and mitigation of climate change.**	
A.1.2.1.1. Policies/Decisions on health options – GHG reduction in transportation	
Questions:	Actions:
*• Does the government plan to transform the transportation system in my city?*	*• Gasoline vehicle tax policies.* *• Making the best decision: changing the way you get around the city.*
A.1.2.1.2. Policies/Decisions on health options – GHG reduction in other daily activities	
Questions:	Actions:
*• How is a responsible consumption policy designed and executed for the territory?* *• How can we design a plan to access local food and thus reduce the carbon footprint in the neighborhood?*	*• Reward policies for homes that consume less energy in the city.* *• Home challenge: reduce the consumption carbon footprint.*

**Box 2:** Examples of stakeholders’ questions and actions using the taxonomy coding system.

A. PROMOTION OF HEALTH AND CLIMATE CHANGE.	
A.1.1. Governance in health and adaptation to climate change.	
A.1.1.1.1. Policies/Decisions on health options – Water protection.	
Questions:	Actions:
*How can we handle wastewater without risks?* *How can we achieve permanent drinking water in the territory?*	*Plans for oxidation ponds.* *Subpoenas/fines for discarding tires in water sources.* *Waste collection to raise awareness for the care of oceans and rivers.* *Legislation and actions to protect moorlands, headwaters and water deposits via the collective protection of ecosystems.*
A.1.1.1.2. Policies/Decisions on health options – Reduction of the impact of floods.	
Questions:	Actions:
*How can we create the infrastructure and education to eliminate mosquito breeding grounds in stagnant water during rainy seasons?* *Which citizen actions would push decision-makers to reduce the probability of floods given the current climate variability?*	*Sewers for draining wastewater.* *Green urbanism.* *Economic incentives for populations that reduce and safely handle waste in floodplains (for example, via taxes, or reducing utility fees).* *Decent plans for population relocation.*
A.1.1.1.3. Policies/Decisions on health options – Heatwave alerts.	
Questions:	Actions:
*How can we design a health surveillance plan to face heatwaves?*	*Planting trees that will increase the local topsoil. Including construction standards with green roofs/walls in legislation.*
A.1.1.1.4. Policies/Decisions on health options – Drought management.	
Questions:	Actions:
*What infrastructure and actions can collect rainwater safely and efficiently in the territories?* *Which local policies help save water?* *Which productive processes can be modified to reduce water use?*	*Planning the distribution of drinking water during crises.*
A.1.1.2.1. Sustainable systems in the interest of life – Water protection.	
Questions:	Actions:
*How can we reduce individual and industrial water footprints?* *Which open information systems will inform us of the quality, quantity and access conditions to drinking water in the territory?*	*Strengthen local capacities to guarantee drinking water.* *Implement domestic technologies to reduce drinking water consumption.* *Domestic and industrial water-recycling systems.*
A.1.1.2.2. Sustainable systems in the interest of life – Reduction of the impact of floods.	
Questions:	Actions:
*Which rainwater collection systems reduces the risk of floods?* *What does an early flood alert system look like?*	*Locally organized collection and recycling of used tires.* *Design and implementation of collection and waste handling systems inside and outside households.* *Construction of adequate housing in non-floodable zones and/or with urban sanitation systems and sustainable drainage.*
A.1.1.2.3. Sustainable systems in the interest of life – Heatwave alerts.	
Questions:	Actions:
*How can territories or their citizens generate an early heatwave alert system?*	*Citizen monitoring of local temperatures.*
A.1.1.2.4. Sustainable systems in the interest of life – Drought management.	
Questions:	Actions:
*Which open information systems informs us of the quality, quantity and conditions of drinking water in the territory?* *What does an alternative citizen plan to face drought risk look like?*	*Domestic and industrial water recycling systems.* *Local identification of alternative drinking water sources.*
**A.1.2. Governance in health and mitigation of climate change.**	
A.1.2.1.1. Policies/Decisions on health options – GHG reduction in transportation.	
Questions:	Actions:
*Which strategies have reduced GHG from transportation in different parts of the world?* *Which advocacy tools impact public policy decisions to implement clean public transportation systems?*	*Urban planning with bicycle lanes and/or exclusive disposal of streets for bicycle riders and pedestrians.* *Raising taxes on vehicles that use fossil fuels.* *Legislation in the interest of life.*
A.1.2.1.2. Policies/Decisions on health options – GHG reduction in other daily activities.	
Questions:	Actions:
*Which daily practices increase/reduce carbon footprint?*	*Generate alternatives for the consumption of goods and services with a guaranteed low carbon footprint.* *Legislation in the interest of life.*
*Continue*	
**Box 2.** Examples of stakeholders’ questions and actions using the taxonomy coding system. Continuation.	
A.1.2.2.1. Sustainable systems in the interest of life – GHG reduction in transportation.	
Questions:	Actions:
*Which massive transportation systems reduce GHG in the territory?*	*Increase walking and the use of public transportation and bicycles.*
A.1.2.2.2. Sustainable systems in the interest of life – GHG reduction in other daily activities.	
Questions:	Actions:
*Which goods/services are produced with low GHG?*	*Recycling PET plastic bottles and organic waste.* *Consumption of home-grown food: a healthy, sustainable, and delicious diet.* *Generate recycling systems, compost and a viable domestic diet.*
**A.2.1. Healthy environments and adaptation to climate change.**	
A.2.1.1.1. Healthy family/home – Water protection.	
A.2.1.1.2. Healthy family/home – Reduction of the impact of floods.	
A.2.1.1.3. Healthy family/home – Heatwave alerts.	
A.2.1.1.4. Healthy family/home – Drought management.	
A.2.1.2.1. Healthy street, block, neighborhood – Water protection.	
A.2.1.2.2. Healthy street, block, neighborhood – Reduction of the impact of floods.	
Question:	Actions:
*How can we prevent the formation of mosquito breeding grounds in stagnant water during rainy seasons?*	*Efficient sewage system.* *Reusing PET bottles.* *Avoiding the accumulation of animal feces that could be dispersed by rain.* *Informative campaigns in small neighborhood stores so people know the importance of recycling.*
A.2.1.2.3. Healthy street, block, neighborhood – Heatwave alerts.	
A.2.1.2.4. Healthy street, block, neighborhood – Drought management.	
A.2.1.3.1. Healthy institutions – Water protection.	
A.2.1.3.2. Healthy institutions – Reduction of the impact of floods.	
A.2.1.3.3. Healthy institutions – Heatwave alerts.	
A.2.1.3.4. Healthy institutions – Drought management.	
A.2.1.4.1. Healthy city - Water protection	
A.2.1.4.2. Healthy city - Reduction of the impact of floods.	
Questions:	Actions:
*How can we prevent the formation of mosquito breeding grounds in stagnant water during rainy seasons?*	*Efficient sewage system.* *Keeping streets clean from waste and debris.* *Garbage containers for waste collection.*
A.2.1.4.3. Healthy city - Heatwave alerts.	
A.2.1.4.4. Healthy city - Drought management.	
A.2.2. Healthy environments and mitigation of climate change.	
A.2.2.1.1. Healthy family - GHG reduction in transportation.	
A.2.2.1.2. Healthy family - GHG reduction in other daily activities.	
A.2.2.2.1. Healthy street, block, neighborhood - GHG reduction in transportation.	
A.2.2.2.2. Healthy street, block, neighborhood - GHG reduction in other daily activities.	
A.2.2.3.1. Healthy institution - GHG reduction in transportation.	
A.2.2.3.2. Healthy institution – GHG reduction in other daily activities.	
A.2.2.4.1. Healthy city – GHG reduction in transportation.	
A.2.2.4.2. Healthy city – GHG reduction in other daily activities.	
**A.3.1. Health knowledge and adaptation to climate change.**	
A.3.1.1.1. Informing and training communities to act and protect health and life – Water protection.	
A.3.1.1.2. Informing and training communities to act and protect health and life – Reduction of the impact of floods.	
A.3.1.1.3. Informing and training communities to act and protect health and life – Heatwave alerts.	
A.3.1.1.4. Informing and training communities to act and protect health and life – Drought management.	
A.3.1.2.1. Transformation of practices via education – Water protection.	
A.3.1.2.2. Transformation of practices via education – Reduction of the impact of floods.	
A.3.1.2.3. Transformation of practices via education – Heatwave alerts.	
A.3.1.2.4. Transformation of practices via education – Drought management.	
A.3.1.3.1. Communal and participative investigation – Water protection.	
A.3.1.3.2. Communal and participative investigation – Reduction of the impact of floods.	
A.3.1.3.3. Communal and participative investigation – Heatwave alerts.	
A.3.1.3.4. Communal and participative investigation – Drought management.	
**A.3.2. Health knowledge and mitigation of climate change.**	
A.3.2.1. Informing and training communities to act and protect health and life.	
A.3.2.1.1. Informing and training communities to act and protect health and life – GHG reduction in transportation.	
A.3.2.1.2. Informing and training communities to act and protect health and life – GHG reduction in other daily activities.	
A.3.2.2.1. Transformation of practices via education – GHG reduction in transportation.	
A.3.2.2.2. Transformation of practices via education – GHG reduction in other daily activities.	
A.3.2.3.1. Communal and participative investigation – GHG reduction in transportation.	
A.3.2.3.2. Communal and participative investigation – GHG reduction in other daily activities.	
**B. PREVENTION OF DISEASE AND CLIMATE CHANGE.**	
**B.1.1. Primary prevention of disease and adaptation to climate change.**	
B.1.1.1.1. Preventing diseases of different age groups – Water protection.	
Questions:	Actions:
*How can we improve access to basic sanitation systems?* *Which human activities pollute rivers more and how does it affect the health of nearby inhabitants?*	*Teaching people how to treat water for consumption.*
B.1.1.1.2. Preventing diseases of different age groups – Reduction of the impact of floods.	
Questions:	Actions:
*How can we prevent the formation of mosquito breeding grounds in stagnant water during rainy seasons?*	*Periodic cleaning of homes to avoid breeding grounds.* *Efficient sewage system. Infographics and videos for education on floods.*
B.1.1.1.3. Preventing diseases of different age groups – Heatwave alerts.	
Questions:	Actions:
*Can high temperatures affect mental health?*	*Plant native trees to adapt to heat waves.*
B.1.1.1.4. Preventing diseases of different age groups – Drought management.	
B.1.1.2.1. Preventing metabolic and cardiovascular diseases – Water protection.	
B.1.1.2.2. Preventing metabolic and cardiovascular diseases – Reduction of the impact of floods.	
B.1.1.2.3. Preventing metabolic and cardiovascular diseases – Heatwave alerts.	
B.1.1.2.4. Preventing metabolic and cardiovascular diseases – Drought management.	
**B.1.2. Primary prevention of disease and mitigation of climate change.**	
B.1.2.1.1. Preventing diseases of different age groups – GHG reduction in transportation.	
B.1.2.1.2. Preventing diseases of different age groups – GHG reduction in other daily activities.	
B.1.2.2.1. Preventing metabolic and cardiovascular diseases – GHG reduction in transportation.	
Questions:	Actions:
*How can I encourage my neighbors to use their cars less and walk or ride bicycles more often?*	*Walk, use public transportation, and ride bicycles more often.*
B.1.2.2.2. Preventing metabolic and cardiovascular diseases – GHG reduction in other daily activities.	
Questions:	Actions:
*What other initiatives, apart from those related to transportation, can improve our health as climate change is mitigated?*	*Reduce the consumption of foods with saturated fats, refined sugars and salt.* *Increase consumption of homegrown vegetables.*

As we mentioned, the matrix works to delimit each research question on public health and climate change or to locate the scope of citizen actions against climate change. This tool is the initial outline with which we hope to obtain a uniform language to understand stakeholders’ ideas in different places and contexts. With a broader view, we hope this taxonomy allows for better communication in health, well-being and climate change issues. Thus, any social actor in any territory can propose and share action ideas, which could be used to evaluate public policy actions, or even to identify the scope of a research project in communities.
